# Myths and Modern Therapeutics

**Published:** 1888-09

**Authors:** F. R. Campbell

**Affiliations:** Professor of Materia Medica and Therapeutics, Niagara University; No. 31 Franklin Street


### MYTHS AND MODERN THERAPEUTICS.

By F. R. CAMPBELL, A. M., M. D.,

Professor of Materia Medica and Therapeutics, Niagara University.

The present is an era unrivaled in the history of medical progress. The microscope, the discoveries of organic chemistry and the improved methods of biological research have apparently revolutionized the science of medicine, and the physician of the present day is prone to regard the practice of his art as being for the first time in history based upon well-established scientific principles. So busy are we with new discoveries, new remedies and new theories, that comparatively few of us are acquainted with even the rudiments of medical history. A few centuries ago European physicians, like those of China at the present day, blindly worshipped the opinions of the Ancients. We of the present age have gone to the opposite extreme, and consider nothing good except what is new. A hundred medicine factories are flooding the country with new drugs, when the majority of physicians do not know the nature of half the remedies in the pharmacopœia. A thousand specialists are swamping the profession with a mass of new books, and new treatments for diseases, made up in the majority of cases of very old ideas. We constantly hear so much about new drugs that I am inclined to think a treatise on "old remedies" would be a very refreshing change. The thoughtful medical man will, however, admit that the human mind has been ever the same; that every age has had its favorite medical theories supported by the ablest thinkers; and the mass of physicians who either ridiculed or pitied the ignorance of their predecessors and told in boastful language of the great improvements and discoveries made in *their* day.

The present is the offspring of the past, as the future will be the child of the present, and I trust it will not be uninteresting to study briefly some of the ancestors of our present medical opinions, especially those whose features have been preserved in the medical practice of modern times. We cannot discuss these theories and notions in proper geneological order. It is very difficult to determine in what instances the discovery of a number of facts has, by a process of inductive reasoning, given rise to a theory, and when ; on the other hand, the rules of practice have been deduced from the pre-existing theory.

It has always been a characteristic of the human race to look upon things which were not clear to their understanding as being of supernatural origin. In the infancy of mankind, as among savage tribes at the present day, we find diseases personified and regarded as evil spirits which had taken possession of the patient. The American Indian medicine-man puts on his fiercest dress and paint and endeavors by horrid noises and grimaces to frighten the disease away. The Chinese doctor in ages past as at the present day, when his patient was racked with pain, pricked the afflicted part with needles to let the offending spirit out, or applied burning cotton to the skin. Thus acupuncture, the moxa and the cautery were introduced into therapeutics. The ancient pagans invoked the aid of higher gods and spirits to expel the offending demon, and we find Apollo Æsculapius invoked by men as wise as Socrates. With the early Jews the same custom was observed, and the great "I Am" was invoked for the cure of all distempers. Then we read in the Second Book of Chronicles: "And Asa in the thirty and ninth year of his reign was diseased in his feet until his disease was exceeding great ; yet in his disease he sought not to the Lord, but to the physicians. And Asa slept with his fathers."

In the New Testament we find Jesus and the Apostles curing diseases and casting out devils, which were about the same thing, according to popular pathology of that day. We find this power of the higher spirit over the lower exemplified throughout the Bible and the history of the Church, though the physician may be interested to know that Celsus and Galen ridiculed the pretensions of the Church Fathers. Even at the present day, when people are found with sufficient faith, *i. e.*, with sufficient ignorance and credulity, diseases of all forms are cast off like old garments when the patient is blest by prayer or touched by the benign finger of the priest. Then we find that the prayer cure, the ancestor of the modern mind cure, of hypnotic suggestion, etc., was in some form or other the earliest of the therapeutic measures. When medical practice had assumed a scientific form, the prayer cure was still influential. Galen believed in charmed amulets; the early Hindoos and Greeks headed their prescriptions with prayers, that no evil spirit might interfere with the action of the drugs; and we still retain the sign of Jupiter, the king of the gods, at the head of our recipes as a relic of this pious practice.

The primitive man, in watching the lower animals, observed that they, too, became ill, and often recovered. From a very natural course of reasoning it was decided that the gods took a special interest in their welfare. From the earliest time it was believed that the gods revealed their will and wisdom to man through the lower animals. The howling of the dog, the flight of the birds, and the disposition of the entrails of the sacred victim were all signs pregnant with revelations from the deities. The goat was sacred to Jove, the ox to Apollo, the dog to Mars, and the hippopotamus and ibis to the god of the Nile. By watching the conduct of these animals when ill, it is said that men obtained their earliest knowledge of remedies. In Egypt especially this idea was very prevalent, for here both gods and sacred animals were so numerous that Herodotus tells us, "It is easier to find a deity than a mortal in Egypt." We are informed by Pliny that the Egyptians learned phlebotomy from the hippopotamus. "For he, finding himself fat and cumbersome on account of continual high feeding, goes forth from the water to the shore, where he has espied a sharp and pointed cane. With this he pricks a certain vein in one of his legs, and thus bleeds himself, making evacuation whereby his body, inclining to disease, is well eased of all superfluous humors." We are also told that the sacred ibis, when ill, fills his long bill with the Nile water and carefully inserts it into the bowel, thereby evacuating the noxious humor, and in this way were first learned the use of clysters. The dog, also, when his stomach contains irritating substances, eats an indigestible grass, which acts as an emetic, thus removing the baleful humor.

From Theophrastus, who lived some time before Pliny, we learn that Melampus (1400 B. C.) observed that goats suffering from phrenzy or insanity, manifested by their lively movements and capers, were in the habit of purging themselves with hellebore, and thus effecting a cure. From this he was led to treat the insane daughters of the King in a similar manner, and effected a marvelous cure. The use of a brisk purge in the treatment of cerebral inflammation is still regarded as excellent treatment.

Not only were there sacred animals, but also sacred plants, and even sacred or rather consecrated minerals, among the ancients. The Egyptians regarded the squill or sea onion growing at the mouth of the Nile as sacred. We are told that Pythagoras wrote a volume on its medicinal qualities. Hippocrates used it extensively, giving it internally, externally, and as a pessary or suppository.

The mandrake, *mandragora officinorum*, was worshipped by the Scandinavians. The ancient Greeks regarded it as a male plant, and called it *anthropomorphon*, from the fancied resemblance of the root to the form of the human body. It was prized by women as a remedy for barrenness, a fact which will sufficiently explain the high value that Rachel of Old Testament fame placed upon a few of these tubers which Reuben had found in the fields. In Genesis XXX. we read: "And Reuben went in the days of the wheat harvest and found mandrakes in the field and brought them unto his mother Leah. Then Rachel said to Leah: 'Give me, I pray thee, of thy son's mandrakes.' And she said: 'Is it a small matter that thou hast taken my husband, and wouldest thou take my son's mandrakes also?' And Rachel said: 'Therefore he will lie with thee to-night for thy son's mandrakes.' And Jacob came out of the field and Leah said: 'Thou must come in unto me, for I have hired thee with my son's mandrakes.'"

Many of the Indian tribes at the present day have sacred plants. Some Mexican tribes, for example, worship the eryodiction or *Yerba Santa*, while some varieties of hydrangea are similarly regarded by northern tribes.

The ancient Greeks looked upon sulphur as sacred, a fact which is demonstrated by the etymology of its Greek term θεῖον, divine. In Homer's Iliad we read that Achilles, in preparing the sacrificial vessel, "Cleansed it in bright sulphur's sacred flame." And in Genesis we find Jehovah raining fire and brimstone on Sodom and Gomorrah. In fact, all substances having a sharp or aromatic odor were regarded as containing spirits powerful for good and grateful to both gods and man. The Egyptians placed these substances about the bodies of the dead, thus discovering their antiseptic virtues, which were applied both in the embalming of the dead and the dressing of wounds thousands of years before men dreamed of the modern theory of antisepsis.

In the history of pharmacy we find that the aromatics were among the first remedies used internally by all nations. They acted on the tormented bowels like a hymn, *carmen*, to the gods, dispelling the evil spirits which racked the body with pain. Hence we find them classed by the old Latin writers as at the present day with carminatives.

Some practitioners, instead of courting the favor of higher deities with pleasant offerings, believed in attacking directly the demon which offended the body. With this object in view, the body was dosed with loathsome draughts, repulsive to every human sense. Urine, human excrement, and drugs like asafœtida, with a noxious odor, were given to drive the disease from the body, much as a boy attempts to smoke a groundhog from his hole. We are informed that the evil spirits causing convulsions will often leave the body when asafœtida is given, thus giving it a reputation for the cure of hysteria, which it still retains.

To the ancient mind all things were occupied by spirits. The rivers, mountains, flowers, minerals, and gems were each the home of some potent demons; and among these divinities there existed sympathies and antipathies. Pliny has an interesting chapter on this subject. He shows how the ivy has a sympathy for the oak; how the birch abhors the maple, and how the crocodile, a word derived from κρόκος, saffron, and, δειλός, afraid, dreads the saffron. Now, it was very natural that the ancients should also believe in the sympathies and antipathies between drugs and disease, thus attaining to something like the modern doctrine of the antagonism between remedies, and remedies and diseases, discussed so ably by Bartholow and Headland.

We learn that the amethyst will prevent intoxication, the hyacinth, hops, and poppy will cure insomnia, the bezoar is a universal antidote for all poisons, while amber is a sovereign panacea for all cerebral affections. Many of those substances were worn about the body as prophylactic charms, much as some of the moderns carry potatoes in their pockets to ward off rheumatism, or wear black ribbons about the neck to prevent throat diseases, In the treatment of the *morbus sacer*, epilepsy, the patient often felt the demon entering some particular portion of the body, and ligatures of colored cord were advised to keep the offender out, a proceeding recommended by some high authorities in the treatment of Jacksonian epilepsy at the present day.

In the remotest antiquity the alchymists busied themselves in extracting the spirits from plants, gems, and minerals, and to their industry we owe the discovery of the essential oils, spirits, tinctures, and the soluble salts of metals. It was argued, *e. g.*, that if the amber as a talisman would cure cerebral affections, the same substance in liquid form would act even more powerfully, and the *oleum succini* was invented, and still retains a place among the cerebral stimulants in the modern pharmacopœia. Many of the alchymists succeeded in extracting spirits from drugs, and kept them imprisoned in bottles, which they carried about the country.

We are told that when some of these spirits were allowed to enter the body, or were inhaled, insensibility and even madness were produced, from which we may infer that some of the anæsthetic ethers were known even at this early day.

The spirit of wine (brandy) was obtained in the tenth century, and was for centuries regarded by many as the long-sought

*elixir vitæ* which would rejuvenate the old, and infuse new life into the body grasped by the hand of death. Monasteries were converted into distilleries of brandy, and even in the present century some of these monastic establishments were devoted principally to the manufacture of liquors and cordials. "Who would believe," says Villanova, "that from wine can be extracted a fluid having neither the color, nature, nor effects of wine! This spirit is called *aqua vitæ*, and abundantly justifies its name, for it prolongs human life." Many were the miracles wrought by brandy; even the dead were raised, the basis for these statements probably being the revival of the patient after being attacked with syncope.

It was believed by the ancient practitioners that the spirits of drugs, charms and talismans acted most powerfully on the parts with which they were brought into direct contact. If applied as ointments and blest with a prayer, they would be most efficacious. From this idea the linimenta, unguenta, cerata and emplastra were developed. In fact, it is to this apparently nonsenical theory that many of the useless plasters of the pharmacopœia owe their existence, for they are certainly inert as far as any medicinal action is concerned. Among these may be mentioned the Emplastra Ammoniaci, Ammoniaci cum Hydrargyro, Arnicæ, Asafœtidæ, Ferri, Galbani, Hydrargyri, and Plumbi, the majority of which are inert and have a place on our shelves only as monuments of the ignorance of our ancestors.

One of the earliest theories influencing the therapeutic application of remedies was the *doctrine of signatures*. It was believed that the deity had so marked plants and animals, that their medical uses could be inferred from their color, form, or some other mark which characterized them. This doctrine is the basis of the Chinese practice at the present day. It was also known to the ancient Hindoos and transmitted by them to the Arabs and mediæval European practitioners. According to this theory, red remedies act principally on the blood, and so cochineal and oxide of iron found a permanent place in the pharmacopœia. Yellow drugs acted chiefly on the yellow bile of the stomach, and turpeth mineral and saffron were addressed to this organ. Ginseng root, having a fancied resemblance to the human body, is regarded as a panacea for all diseases, and enters into nearly every Chinese prescription. In fact, they believed in a homœopathy of forms. The ancient priestly schools that believed in the demoniac pathology were divided as to the proper way of managing the devils, and might be classed as sympathists who believed in giving remedies pleasing to the disease, and antipathists who would give remedies offensive to the disease, the former resembling the homœopathists and the latter the allopathists, if there ever were such a school of practitioners. Even Hippocrates was influenced by these schools, for he says: "Some diseases are cured by similars and others by dissimilars." And might they not both have been right? For at least half the drugs we employ, given in small doses, have directly opposite effects from the same drugs given in large doses. When any of the organs or fluids of the body were diseased, it was believed that these could be cured by giving the essence of the same part taken from another man, or one of the lower animals. If the blood was diseased or deficient, blood was administered. If the bile was affected, bile was given; affections of the stomach called for an essence of the stomach of some of the lower animals; diseases of the head required a preparation of brains, while impotence was cured by giving frog's sperm, spirit of human testicle, or castor and musk, which were supposed to be the male element of the beaver and musk deer. Looking over our own materia medica one will be struck with the prevalence of a similar idea. One manufacturer gives a concentrated preparation of ox brains known as kephaline lit. ext. or alkaloid of head. Dried beef blood is recommended for anæmia. And even in the pharmacopœia will be found *fel bovis inspissatum*, *pepsinum castoreum* and *moschus*, although the last two are no longer recommended to restore the sexual powers. When we consider these things, we have only advanced a step from those who recommended human skull for rickets, and dog's teeth for the cure of hydrophobia.

Astrology, also, has had its influence upon medical practice. And indeed there seems to be some real connection between the movements of the heavenly bodies and the phenomena of human life. The well-regulated woman menstruates once in each lunar period, and in her native primitive state bears a child with each revolution of the earth in its orbit. When the sun is in *scorpio* or *leo*, diseases of the bowels prevail; in *aquarius* and *pisces* the respiratory organs are liable to be affected; and when *Sirius* sheds his lurid rays upon the earth, it is still believed by man that dogs and men go mad. We are not surprised that the ancient physicians traced a close relation between the motion of the stars and the diseases of man. Some plants were only efficacious if gathered under particular constellations and phases of the moon, and we now recognize the fact that the quantity and quality of the active principles contained in vegetable products varies with the age of the plant. Astrology, first cultivated by the ancient Hindoos and Egyptians, was adopted by Arabic sages, and became associated with alchymy and medicine. Each of the metals was regarded as sacred to the sun, or moon, or to one of the planets, and we find the metals designated in the writings of the alchymists by their astrological signs. Each portion of the body was under the direct control of some constellation or heavenly body. The sun presided over the heart, the fountain of life, and each zodiacal sign controlled some portion of the body, as represented in our almanacs of to-day.

*Gold* was the sun's metal, and was called by the alchymists *sol.* It acted upon the heart, and thus produced life. We read in Chaucer—

> "For gold in physick is a cordiale,
> Therefore he lovede gold in speciale,"

—all remedies acting on the heart being known as cordials.

The *aurum potabile* of the alchymists was probably the officinal salt, *auri et sodii chloridum*, and was used with much success as a tonic and alterative. Bartholow recommends gold as a valuable alterative in the treatment of syphilis, and as a tonic in the treatment of impotence.

*Silver* was sacred to *Diana* or the moon, and was called *Luna*. The moon presided over the head and its diseases. For example, the *morbus sacer*, epilepsy, and all forms of insanity, were due to the malign influence of the moon. Very naturally the moon's metal in solution, *argenti nitras*, or lunar caustic, was suggested as a cure for all moon-stricken patients. This treatment of epilepsy, so absurd in its origin, is still recommended in many of our best works on therapeutics, and I have myself seen a patient made as blue as a whetstone by the extensive use of nitrate of silver for epilepsy.

Mars presided over the blood, and *iron* was the war god's metal. Hence, when the blood was diseased or deficient, the *martial* or ferruginous preparations were uniformly administered.

*Mercury* presided over the soul and animal spirits. People of a lively disposition were said to be of a mercurial temperament. The ancients located the soul and mind in the liver and diaphragm (φρήν). If the liver was diseased, men became melancholy *i. e., black bilious*, and mercury was indicated to infuse new spirits and cure the liver. The use of the mercuries in the therapeutics of hepatic diseases is not much more intelligent at the present day, for we do not know whether biliousness is an insufficiency or excess of bile; whether calomel, the preparations preferred by the ancients, stimulates or depresses the activity of the liver. In fact, we give calomel much as our alchymistic ancestors did, because it is a beautiful (καλός) substance for black (μέλας) bile. Paracelsus probably discovered the antisyphilitic action of mercury, and claimed that he possessed in it the true elixir of life. That he was somewhat mistaken is demonstrated by the fact that he died at the age of forty-eight.

Venus very properly presided over the sexual organs, and copper, *cuprum*, first obtained on her island, κυπρός, was her sacred metal Cardan advised his patients afflicted with love-melancholy "to piss through a copper ring," as the expression is translated by Burton. Sulphate of copper was used locally in the treatment of urethritis and venereal ulcers, and the acetate of copper was given internally to strengthen the sexual powers.

Saturn presided over the abdomen, and *lead* was the saturnine metal. The principal disease of the bowels known to the ancients were the fluxes, and the lead salts were administered to cure them.

Within the last few years the theory of metallotherapy has been revived in a new form by such men as Barcq, Petit, Westphal, Morton, and Bartholow, who believe that by placing certain metallic discs in contact with a patient affected with neuralgia or paralysis, wonderful curative effects may be accomplished. After carefully comparing modern metallotherapy with the theory of the astrological alchymists, I am inclined to regard the latter as by far the more plausible. At least it may be said that their notions have furnished a much greater number of valuable contributions to the therapeutic art.

The alchymists believed that there were male and female metals. This doctrine was held by the Hindoos and Egyptians, and may even be traced in ancient Greece. The word arsenic, used by Hippocrates, means, literally, masculine, from *ἄρσην*, a male. It was believed that the administration of male metals would increase strength, and in man increase the sexual powers. The use of female remedies would render men beautiful, but effeminate. Among the Styrians at the present day, arsenic is habitually used by the men as a tonic, they believing that it greatly increases strength and endurance. In some of the epidemic nervous diseases a few centuries ago, it was observed that the women, especially virgins and widows, were chiefly affected, and as a curative measure arsenic was recommended as the most certain method of introducing the male element into their system, although marriage, and even sexual intercourse without marriage, was advised by some physicians. Here we have arsenic clearly recommended as a nerve tonic, and used much at the present day for a specific in the treatment of chorea.

Antimony, *stibium*, was a female metal, and was anciently employed as a cosmetic. This metal, given to men, would weaken the sexual powers, and it may have been this idea that induced Basil Valentine to give it to the monks under his charge, for these gentlemen, we are informed, often found it very difficult to keep their passions within due bounds. We learn, however, that he found it "not good for monks," and hence he called it *antimonium*.

The first really scientific system of medicine was based upon the humoral pathology. Hippocrates believed, as the Hindoos and Egyptians had believed before him, that derangements of health were largely due to disproportions of the four humors: blood, phlegm, yellow bile, and black bile. That this theory has made a great impression upon modern practice, is evinced by the etymology of many of the words in common use at the present day. *Dyscrasiæ* are literally bad mixtures, from δύς, bad, and κεράννομι, to mix. Idiosyncrasy is one's own (ἴδιος) mixing together (συγκρασίς). A cachexia is the possessive (ἕξις) of bad (κακός) humors. The etymology of the Latin temperament and distemper also take us back to the humoral pathology. Emetics were given to carry off the excess of yellow bile and phlegm, and we still use emetics in small doses to act as expectorants. Cathartics carried down the black bile. Blood was removed by leeches, phlebotomy, and arteriotomy. Blisters and setons were used to draw the phlegm or inflammatory humor from the affected part, and we find Villanova advising that lunatics acquire gonorrhœa, as in this way the semen and phlegm could be drawn away from the head. We might trace the history of numerous other abandoned medical theories, but the story would be similar in each.

In conclusion, I will say a word about the recent suggestion of Dr. Gouley, who proposes to revise the nomenclature of disease, because many of the names are based upon erroneous ideas. He would invent a new set of names, which will express exactly the pathological condition. We must criticise the attempt as premature and useless. Who knows that we have discovered even the rudiments of the true pathology? Were not the *astrologists*, the *humoralists*, and the *solidists* of old equally as certain as the modern bacteriologists, that they had discovered the true nature of disease? Who can say that some writer in years to come will not ridicule those nineteenth-century doctors who believed that micro-organisms, like the demons of old, produced diseases in man, that the curative art consisted in germ poisoning, and acted like the Irishman, who, having swallowed a potato-bug, took a dose of Paris green to kill him? Will not our successors be much amused at the myths believed by us, when they learn that, like the colic specialist of Laputa, we filled patients' bowels with stinking gas to cure the lungs, that we believed digitalis would strengthen a weakened heart, and that quinine would cure all intermittent affections?

If the nomenclature of disease were revised, it would require remodeling within a few years, for medicine is not an exact science, and never will be. The introduction of a new nomenclature would make our literature to a degree unintelligible. In medicine, as in other fields of human thought, we should accept the poet's advice—

> "Watch the wheels of nature's mazy plan,
> And learn the future by the past of man."

No. 31 Franklin Street.

